# The Calm Before the Storm: Using In Situ Simulation to Evaluate for Preparedness of an Alternative Care Hospital During the COVID-19 Pandemic

**DOI:** 10.1017/dmp.2021.80

**Published:** 2021-03-25

**Authors:** Gianna Petrone, Linda Brown, William Binder, Sonya Naganathan, Scott Pasichow, Heather Rybasack-Smith, Cathy E. Duquette, Dana R. Palka, Andrew N. Musits

**Affiliations:** 1 Department of Emergency Medicine, Warren Alpert Medical School, Brown University, Providence, RI, USA; 2 Departments of Emergency Medicine and Pediatrics, Warren Alpert Medical School, Brown University, Providence, RI, USA; 3 Global Emergency Medicine Fellow program, Department of Emergency Medicine, Warren Alpert Medical School, Brown University, Providence, RI, USA; 4 Department of Emergency Medicine, Southern Illinois University School of Medicine, Springfield, IL, USA; 5 Quality and Safety, Nursing, Lifespan, Providence, RI, USA; 6 Clinical Operations, Adult Ambulatory Services, Rhode Island Hospital, Providence, RI, USA; 7 Department of Emergency Medicine, Warren Alpert Medical School, Brown University, Providence, RI, USA

**Keywords:** disaster planning, emergency preparedness, field hospital, patient simulation

## Abstract

**Objectives:**

Coronavirus disease (COVID-19) has been identified as an acute respiratory illness leading to severe acute respiratory distress syndrome. As the disease spread, demands on health care systems increased, specifically the need to expand hospital capacity. Alternative care hospitals (ACHs) have been used to mitigate these issues; however, establishing an ACH has many challenges. The goal of this session was to perform systems testing, using a simulation-based evaluation to identify areas in need of improvement.

**Methods:**

Four simulation cases were designed to depict common and high acuity situations encountered in the ACH, using a high technology simulator and standardized patient. A multidisciplinary observer group was given debriefing forms listing the objectives, critical actions, and specific areas to focus their attention. These forms were compiled for data collection.

**Results:**

Logistical, operational, and patient safety issues were identified during the simulation and compiled into a simulation event report. Proposed solutions and protocol changes were made in response to the identified issues.

**Conclusion:**

Simulation was successfully used for systems testing, supporting efforts to maximize patient care and provider safety in a rapidly developed ACH. The simulation event report identified operational deficiencies and safety concerns directly resulting in equipment modifications and protocol changes.

## Introduction

As of early February 2021, about 106 million cases of coronavirus disease (COVID-19) have occurred worldwide, with approximately 27 million cases reported in the United States.^1,2^ While this disease was first encountered in Wuhan, China, it has rapidly spread throughout the world, causing a global pandemic. As the number of cases exponentially increases, so does the need for preparedness. Hospital systems have recognized the need to increase inpatient and critical care bed capacity. In order to meet this demand, many states have developed alternative care hospitals (ACHs), more commonly referred to as *field hospitals*.

Health care systems in the state of Rhode Island were asked to partner with the state and other agencies in creating 3 ACH, with the largest having a 600-bed capacity. This ACH is located in the exhibit hall of a convention center. Over a few short weeks, a building that once housed sporting events and concerts was transformed into a hospital. The design of the ACH includes 6 wards consisting of 3 to 6, 24-bed pods each, a 12-bed transition pod for decompensating patients requiring a higher level of care at the hospital system’s academic facility, and a resuscitation room for patients requiring intubation or more aggressive resuscitation measures.

Many factors are considered when building an ACH; however, patient care, provider, and staff safety remain the highest priorities. In order to perform systems testing, an *in situ* simulation session was developed. The primary goal of this session was to identify areas in need of improvement and suggest changes prior to the facility opening its doors to patients. This might include equipment, flow, protocols, communication pathways, and training priorities. Simulation is a well-established tool to accomplish these objectives.^3^ Previously, simulation has successfully been used for process and safety evaluation^4^; however, the development of an ACH for COVID-19 poses unique challenges. The most notable challenge in responding to a pandemic is the limited preparation time available in anticipation of rapidly opening for patient care. Other challenges include working with multidisciplinary providers in an unfamiliar work environment, the necessity of infection prevention practices, limited resources, and the development of ACH-specific protocols. The methodology described provides site administrators and medical directors the opportunity to uncover potential shortcomings and prevent patient safety issues prior to their occurrence.

## Methods

Four scenarios were developed by an emergency medicine faculty member with experience in simulation and medical education using input from nursing and physician leadership. Scenarios were designed to represent both common clinical situations, as well as low frequency, high acuity events that may arise in the ACH. The scenarios included acute hypoxic respiratory failure, cardiac arrest, fall in bathroom, and staff member syncope. Emergency medicine trained physicians, critical care and medical-surgical trained registered nurses (RNs), and an ambulatory medical assistant (functioning as an inpatient certified nursing assistant [CNA]) were used as the clinical care team in these scenarios. The learning objectives for each scenario can be found in Table 1.

**Table 1.** Learning objectives

Acute Hypoxic Respiratory Failure	Cardiac Arrest	Bathroom Fall	Staff Member Syncope
Identify barriers in responding to respiratory decompensation in the ACH.	Identify barriers in responding to a code blue in the ACH.	Identify barriers in responding to a fall in the ACH.	Identify barriers in responding to a staff emergency at the ACH.
Assess equipment readiness to treat a patient requiring non-invasive ventilation or intubation.	Assess equipment readiness to treat cardiac arrest.	Assess equipment readiness to treat a patient with hypoglycemia or seizure.	Assess equipment readiness to treat a staff member with syncope.
Discuss criteria for moving a patient to the transition pod.	Discuss patient disposition and transfer criteria.	Discuss criteria for moving a patient to the transition pod.	Discuss infection prevention plan for a staff member requiring assistance in the hot zone.
Review effectiveness of communication infrastructure during patient crisis.	Review effectiveness of communication infrastructure during patient crisis.	Review effectiveness of communication infrastructure during patient crisis.	Review effectiveness of communication infrastructure during patient crisis.
Discuss patient disposition and transfer criteria.		Discuss patient disposition and transfer criteria.	Discuss documentation for staff care/emergencies.

*Note*: ACH = alternative care hospital.

Prior to the simulation, participants were assigned the following roles: pod physician, pod nurse, pod CNA, transition pod/resuscitation room physician, and transition pod nurse. These roles were chosen to be consistent with staffing patterns at the ACH. A multidisciplinary group of 15 observers, including physicians, nurses, infection prevention specialists, and pharmacists, acted as observers and were provided debriefing forms (see Appendix A) to complete during and after the simulation. Participants did not have access to the scenarios or debriefing forms prior to the start of the session.

The debriefing forms were developed to compile suggestions and comments in regard to communication, team roles/responsibilities, and logistical concerns, including personal protective equipment (PPE), equipment, protocol modification, and overall readiness. A large group of stakeholders were also present, including various administrators (ACH medical director, health system chief nursing executive, and ACH logistics coordinator), members of the supply chain, emergency medical services (EMS), and the National Guard. The large group was included in the debriefing and discussion; however, they did not complete observer forms.

The participants and observers were oriented to the facility and available equipment prior to the simulation. A high technology simulator was used for the cardiac arrest, hypoxic respiratory failure, and bathroom fall cases, while the staff member syncope case used a standardized patient. A simulation operations specialist was on-site to assist with operations.

Each scenario concluded with a debriefing session summarizing the case, discussing challenges, and identifying areas for potential improvement. Debriefing forms were completed by the observer group and collected at the end of the session. The larger group was also present for the debriefing. Their comments and suggestions were recorded by a designated member of the simulation team. The comments and suggestions were compiled and organized according to area of focus: logistics, communication, team roles, and responsibilities. The information was then distributed to various areas of leadership for further evaluation and utilization.

## Results

The simulation event, including debriefing, concluded after 2.5 hours; 15 participants were present in the observation group and were responsible for real-time evaluations, as well as debriefing at the end of the simulation. These scenarios were instrumental in determining the final facility protocols and identified unforeseen issues that had not been readily identified during meetings or tabletop exercises. The compiled comments and suggestions from the debriefing forms are shown in Table 2.

**Table 2.** Debriefing comments

Acute Hypoxic Respiratory Failure		
Area of Concern	Identified Issue	Proposed/Implemented Solutions
Logistics: PPE, equipment, overall readiness	Importance of removal of all extraneous furniture/equipment from room to allow for stretcher access to the patient	Pod team should remove any unnecessary equipment from the patient care area to allow for improved patient access.
	No slide board available for transfer	Response team to radio for equipment and/or consider emergency medical services assist
	No oxygen tank on stretcher	Each stretcher to be fitted with oxygen tank
	Patient bed does not rise, requiring lifting of the patient.	Response team to communicate need for extra assistance for lifting the patient
	Need for clear guidelines for when patients should be moved to the transition pod and/or resuscitation room	Updated protocols for the decompensating patient
	Transition staff should be in maximum PPE due to risk of aerosolizing procedures.	Transition staff to remain in maximum PPE within the resuscitation room due to risk of aerosolizing procedures
	Clarification on whether using a non-rebreather mask is aerosolizing	Consultation with infection control
	Additional supplies needed for the resuscitation room: supply cart with syringes and needles, suction capability, viral filters for all bag valve masks, positive end expiratory pressure valves, Glidescope® covers	Added to resuscitation room inventory
	Pharmacy requests Omnicell® to replace Pyxis® in the resuscitation room that will include vasopressors as well as other emergent medications not available in the Pyxis®.	Larger Omnicell® with additional medications moved to the resuscitation room
	How to accommodate multiple patients in the resuscitation room. What documentation is being used in the resuscitation room?	Deemed to be unlikely event, however, will space patients within the resuscitation room as needed. Documentation will be performed using the emergency medical record.
Communication	Signage in the resuscitation room on how to contact pharmacy and clear communication plan for “push to talk” devices	Signage placed in the resuscitation room
	Possibility of an overhead announcement for “Code Transfers”	Consider a dedicated channel on push-to-talk devices for these announcements.
Team Roles and Responsibilities	What is the team composition needed in the resuscitation room? How will this change if there is more than 1 patient? Clarification is needed.	One resuscitation room RN, 1 resuscitation room physician, and 1 resuscitation room medical assistant will be present at all times. Resuscitation room staff will have to call for extra assistance on an as-needed basis. Pod physicians and/or pod RNs may respond to the resuscitation room after donning appropriate PPE as needed.
	In what situation can EMS be accessed to assist?	In the event of multiple patients, lift assist, extra equipment
	Is there a designated runner for the resuscitation room?	Review of ancillary staffing numbers
	What staff will have access to the Omnicell®?	Nursing

Cardiac Arrest		
Area of Concern	Identified Issue	Proposed Solution
Logistics: PPE, equipment, overall readiness	No cardiopulmonary resuscitation until maximum PPE. How best is this facilitated if arrest occurs outside of the transition area?	Should all providers wear maximum PPE? Team in pod could place pads/defibrillate.
	No backboard for CPR	Backboard attached to code cart
	No CPR stool	Stool placed in resuscitation room
	Need clock with second hand in resuscitation room for documentation	Clock mounted in resuscitation room
	Need for intraosseous drill in resuscitation room	Intraosseous drill stored in resuscitation room
	Medications including vasopressors should be easily accessible in resuscitation room	Omnicell moved to resuscitation room
	Need for body bags	Body bags stored in resuscitation room and on resuscitation stretcher
	Consider using a LUCAS® device for compressions.	Consider ordering a LUCAS® device.
	Where is the morgue?	Consider a designated area to relocate bodies until they are moved from the site.
Communication	What is the communication plan for codes and transfers?	Consider overhead announcement.
	Who contacts the patient’s family?	Consider designating 1 provider to contact family.
Team Roles and Responsibilities	Could there be a CPR team already in maximum PPE to start CPR? Can we use EMS providers?	Develop an RRT or CPR team in maximum PPE to ensure that resuscitation is not taken away from their patients.
	Who cares for patients when the care team is pulled from the pod or resuscitation room?	Implementing an RRT/CPRteam will eliminate this issue.

Fall in the Bathroom		
Logistics: PPE, equipment, overall readiness	Cervical collar and board needed for transfer	Stock cervical collars and backboards
	Need to identify patient and room location when out of pod	Color coded wristband to identify patient, ward, and bed
Communication	Early communication to EMS personnel on-site to assist with moving/extricating patient if needed	Develop EMS protocol for assisting within the ACH.
	One channel on “push to talk” device for all emergencies	Suggestion communicated to responsible team
Team Roles and Responsibilities	Clear guidelines are needed on when patients can use the bathroom or respite area.	Allowed to travel per nurse’s discretion
	Discussion that all patients in bathroom should have staff member with them as no call alarms are available	Confirmed as guideline
**Staff Member Syncope**		
Logistics: PPE, equipment, overall readiness	Staff member was moved to the transition pod.	Unless CPR is necessary, the staff member should be moved to the donning/doffing area located by the EMS entrance and transferred by EMS to the hospital.
	May want to avoid moving to the transition pod, as this is the highest risk area of exposure to the new coronavirus for the “negative” staff member (now patient)	Move staff member to the donning/doffing area.
	Further discussion of transfer device needed for larger patients	Consider stocking bariatric slide boards or having a “lift team.”
Team Roles and Responsibilities	Need for early EMS role in these patients	Consider protocol development for EMS involvement.
	Process for covering the downed provider’s assignment	Development of a provider down coverage protocol

*Notes*: ACH = alternative care hospital; CPR = cardiopulmonary resuscitation;

EMS = emergency medical services; PPE = personal protective equipment; RRT = rapid response team.

GlideScope®, Verathon®, Bothell, WA, USA

Pyxis®, Becton and Dickinson Company, Franklin Lakes, NJ, USA

Omnicell®, Omnicell, Mountview, CA, USA

LUCAS®, Stryker Medical, Lund Sweden, USA

As a result, some changes were implemented almost immediately, including the addition of an Omnicell^®^ with additional capacity into the resuscitation room, allowing a pharmacy to stock a wider scope of emergency medications. Nursing supervisors were granted access to the Omnicell to assist in resuscitations. A doffing station was added at the ambulance egress to the facility to assist in the care of uninfected staff who may need transport to the hospital. A list of necessary resources and equipment was generated for which the administration immediately began to address. Revisions to current protocols began, including cardiopulmonary resuscitation (CPR) management, criteria for transfer to the transition pod, and the development of a CPR/rapid response team (RRT).

## Discussion

The purpose of this simulation was to focus on protocol development and systems testing for an ACH. While well-developed and tested protocols are frequently created and modified within a hospital system, they are not directly applicable to the ACH setting due to its dynamic and atypical environment. A multi-disciplinary *in situ* simulation offers a collaborative and constructive mechanism to gain insight into potential conflicts and safety issues, and to identify and implement changes prior to the opening of the ACH.

Several elements of the *in situ* simulation process were identified as being critical to the success of this program. In addition to having an experienced and interprofessional team, success was attributed to establishing psychological safety, a team with clear roles, and a debriefing plan.

### Psychological Safety

Participants and their roles were established prior to the start of the session during the pre-briefing. Importantly, we specified that the purpose of the session was not to evaluate the clinical capabilities of the providers and staff, but to investigate and assess systems and processes. Removing the aspect of clinical evaluation alleviated some of the pressure from the individuals participating in the simulation and established psychological safety. It should also be noted that this was again acknowledged during the debriefings. In order to make the scenarios as realistic as possible, the participants were assigned their currently employed role; for example, a physician was assigned as the pod physician. The observer group was interdisciplinary and composed of physicians, nurses, pharmacy, and infection prevention personnel, each offering their unique perspective.

### Team With Clear Roles

Completing a simulation assessment is best accomplished with a dedicated and experienced simulation team. Our simulation team included 3 experienced simulation faculty. One faculty member served as the voice of the patient and provided case information, 1 led the debriefing, and 1 was dedicated to observation and note-taking. Three simulation operation specialists were present to operate the high technology simulator, reset the physical space, and perform audiovisual documentation. As this is a collaborative team effort, the roles may vary, based on the scope of the project, resources, and personnel available.

### Debriefing Process

A well-facilitated debriefing is instrumental in encouraging discussion, knowledge sharing, and identifying areas in need of improvement. For this session, with many participants and observers, it was particularly important to briefly and factually recount the series of events in the simulation. One participant was asked to briefly summarize the case at the beginning of the debriefing. If any phone calls were initiated, the participants were asked to summarize the nature of the communication. These 2 steps allowed for all observers and participants to establish a shared mental model of the simulation case. Next, the simulation participants were given the opportunity to comment on things that went well or posed specific challenges. Finally, the discussion was opened to all present to share observations and suggest solutions to problems identified. It should be noted that there is no single correct way to facilitate a debriefing, as many debriefing frameworks work well for interdisciplinary simulations.^5^

One of the common themes in each of the scenarios involved transporting a patient to a higher level of care within the facility. Many of these are reported in the debriefing comments for the hypoxic respiratory failure scenario. This was the first simulation case and required an extended debriefing. This is helpful to recognize in regard to time management of the session. Facilitators should recognize that the later scenarios will debrief more rapidly, as it is unnecessary to reiterate points previously discussed in a prior debriefing.

### Limitations

It should be recognized that without the cooperation and recognized value from the administration and leadership, this project would not be possible. Full support was provided by the health system chief nursing executive, ACH medical director, ACHF nursing director, and the ACH logistics team.

Every ACH fills a specific niche, experiences unique challenges, and has different resources. While there are certainly common themes identified in this study among various ACH, many of our findings are likely unique to our facility. Therefore, the recommendations in the results section are not prescriptive, but rather demonstrate the granularity of outcomes from this process.

The physicians participating in the simulation were all emergency medicine trained. Emergency medicine physicians are a valuable resource when discussing needs of an ACH and will mostly be staffing the transition pod rather than the wards. Wards will likely be staffed by physicians from other specialties with less experience in critical care, or underutilized providers due to the pandemic. In this ACH design, this includes physicians who typically work in an outpatient or surgical setting and are not usually involved with inpatient medicine. If other specialties were included in the future simulation assessments, additional recommendations might be uncovered. The unique background and difference in training and daily practice of these physicians offer a different perspective. Another challenge was dealing with COVID-19-related restrictions on gathering size and social distancing requirements, limiting the number of people able to participate in the simulation.

## Conclusion

The unpredictable nature of this pandemic has increased demands on the health care system. While the development and implementation of the ACH will help alleviate the burden, these have created a new set of obstacles. *In situ* simulation can be an effective tool for systems testing and evaluating for preparedness during the development of an ACH. We conclude that this study was successful in identifying and resolving possible safety issues prior to the facility opening.
